# Integrative Phosphoproteomic Profiling Reveals Stage‐Specific Signalling and Metabolism in Equine Melanocytic Neoplasm

**DOI:** 10.1111/vco.70070

**Published:** 2026-04-16

**Authors:** Paitoon Srimontri, Amornthep Kingkaw, Nawarus Prapaiwan, Rangsima Sujittosakul, Nichapat Iamkaewprasert, Jiraschaya Piputwat, Puetta Isama‐al, Thanutchanok Munkongdee, Thanapon Chotikaprakal, Petchpailin Yanyongsirikarn, Narumon Phaonakrop, Sittiruk Roytrakul, Wanwipa Vongsangnak, Parichart Tesena

**Affiliations:** ^1^ Department of Clinical Science and Public Health, Faculty of Veterinary Science Mahidol University Nakhon Pathom Thailand; ^2^ Kasetsart University International College (KUIC) Kasetsart University Bangkok Thailand; ^3^ Faculty of Veterinary Science Mahidol University Nakhon Pathom Thailand; ^4^ Biochemistry Unit, Department of Physiology, Faculty of Veterinary Science Chulalongkorn University Bangkok Thailand; ^5^ Equine Clinic, Prasuarthon Small Animal Hospital, Faculty of Veterinary Science Mahidol University Nakhon Pathom Thailand; ^6^ Functional Proteomics Technology Laboratory, National Center for Genetic Engineering and Biotechnology, National Science and Technology Development Agency Pathum Thani Thailand; ^7^ Department of Zoology, Faculty of Science Kasetsart University Bangkok Thailand; ^8^ Omics Center for Agriculture, Bioresources, Food, and Health Kasetsart University (OmiKU) Bangkok Thailand

**Keywords:** equine melanocytic neoplasm, grey horses, phosphoproteomics, PI3K‐Akt signalling, Wnt/β‐catenin pathway

## Abstract

Equine melanocytic neoplasms (EMN) are aggressive tumours characterised by high metastatic potential and limited therapeutic options available. However, the molecular mechanisms underlying their progression remain poorly understood. This study therefore presents the integrative phosphoproteomic analysis of EMN tissue, with the aim of elucidating stage‐specific alterations in signalling pathways and metabolism. Nineteen tissue samples from grey horses were categorised as normal‐stage (*n* = 6), early‐stage EMN (*n* = 7), and severe‐stage EMN (*n* = 6) and subjected to in‐depth analysis using liquid chromatography–tandem mass spectrometry (LC–MS/MS). A total of 2035 phosphoproteins were identified, of which 219 were differentially expressed across the disease stages. Interestingly, early‐stage EMN showed dysregulation of inositol phosphate metabolism and activation of the PI3K‐Akt pathway which involved INPP5F and PKN2. In severe‐stage EMN, upregulation of SYNJ1, STRN4 and VIM indicated enhanced membrane trafficking, cytoskeletal remodelling, and MAPK signalling. Additionally, ASPM and GNAO1 upregulation reflected heightened proliferation and altered Rap1 signalling, while UBR5 dysregulation suggested aberrant protein homeostasis. Metabolic reprogramming was also noticed, with elevated TKT and GAPDH expression supporting glycolysis and NADPH production. Observably, the severe‐stage EMN exhibited a higher expression of Dickkopf‐3 (DKK3) which suggests a role in aberrant Wnt/β‐catenin activation and tumour progression. These findings reveal stage‐specific molecular mechanisms in EMN pathogenesis and highlight potential biomarkers and therapeutic targets for equine melanoma.

## 1 Introduction

Equine melanocytic neoplasm (EMN) represents a significant health issue in aging grey horses involving melanocyte proliferation that results in tumours ranging from benign to malignant [1]. This neoplasm commonly presents as solitary or multiple masses, predominantly in the perianal region, tail base and head, substantially affecting the quality of life and welfare of affected equines [2]. Although the genetic predisposition linked to grey coat colour is a well‐established risk factor, the specific molecular mechanism driving the pathogenesis, progression, and metastasis of EMN remains poorly understood [3]. EMN encompasses a spectrum of melanocytic proliferative disorders in horses, ranging from benign melanocytic tumours to malignant melanomas. These tumours arise from melanocytes, the pigment‐producing cells of the skin and other tissues [4]. The most common form is associated with the grey coat colour phenotype, where progressive accumulation of melanocytes leads to the development of tumours, often in older grey horses. Histologically, EMN may exhibit a spectrum of cellular atypia, mitotic activity, and invasive characteristics, corresponding to the variable clinical behaviours [5]. Previous studies have investigated the genetic and proteomic factors involved in EMN [1, 6].

While polymorphisms in *STX17* are recognised as the main cause of the grey coat phenotype and increased EMN susceptibility, the molecular mechanisms governing neoplastic transformation remain unclear [3]. Other genes, including *ASIP*, *MC1R* and *TYRP1*, which regulate melanocyte function, may also contribute to EMN pathogenesis. Proteomic analysis has revealed disrupted pathways in EMN, especially those related to cell cycle regulation, signal transduction and extracellular matrix remodelling [6]. Differentially expressed proteins identified between normal grey skin and EMN tissues across stages could serve as biomarkers for early diagnosis and disease monitoring [7]. Notably, matrix metalloproteinase 1 (MMP1) was consistently upregulated in EMN, suggesting a role in tumour progression and therapeutic potential [8]. Additionally, the proteomics of serum and faeces have identified candidate proteins linked to melanogenesis, melanoma progression, metabolism, cellular stress and inflammation [6]. Although few studies have investigated EMN phosphoproteomics, protein phosphorylation plays a crucial role in signal transduction and cellular processes. Recent findings by Vinijkumthorn and colleagues highlight the potential of non‐invasive samples to detect phosphorylation changes linked to early‐stage EMN [9]. However, phosphoproteomic analysis across all disease stages using tissue biopsies remains uncharacterised.

Given these challenges, this study aimed to unravel the complex molecular mechanisms underlying EMN pathogenesis by integratively characterising stage‐specific phosphoprotein alterations in tissue biopsies. To achieve this, phosphoproteins were enriched from tissue samples and analysed using LC–MS/MS. The resulting phosphoproteomic data were subjected to quantitative analysis and functional annotation using bioinformatics tools and databases. By integrating these phosphoprotein alterations in response to stage‐specific EMN, this study provides novel insights into the molecular mechanisms driving EMN pathogenesis and identifies potential biomarkers for further disease monitoring and therapeutic intervention.

## 2 Materials and Methods

### 2.1 Ethical Statement

This study was conducted in accordance with ethical guidelines and approved by the Institutional Animal Care and Use Committee (IACUC) of the Faculty of Veterinary Science, Mahidol University, Thailand (approval number: MUVS‐2024‐02‐09). Informed consent was obtained from all horse owners prior to participation. No cell lines were used in this study, which relied on the direct analysis of equine tissue biopsy samples; therefore, cell line validation was not applicable.

### 2.2 Grey Horse Signalment

Nineteen tissue biopsy samples of grey horses with or without the appearance of melanomas were included in the study. The experimental horses comprised of various breeds including Mixed breed (*n* = 7), Australian Warmblood (*n* = 7), Arabian (*n* = 3) and pony (*n* = 2). Within this sample size, 9 geldings and 10 mares were included in the study. The median age of grey horses in the normal group was 5 years (range of 4 to 13 years). Grey horses in the early EMN group were 14 years, and grey horses in the severe EMN group were 14 years. In addition, the median body weight of grey horses with the normal group was 432 kgs (range of 330 to 506 kgs), grey horses with early EMN group were 532 kgs (range of 260 to 646 kgs) and grey horses with severe EMN group were 540.5 kgs (range of 450 to 584 kgs) as shown in Table 1 and Supporting File S1. Vital signs included heart rate, respiratory rate, gut sound, mucous membrane, rectal temperature and the position of the melanomas of each horse were recorded. The EMN were categorised into three groups: normal‐stage, early‐stage EMN, and severe‐stage EMN [6, 7, 9, 10]. In brief, the horses without any melanomas were classified as the normal‐stage group or grade 0. The horses, which have merely nodules with a diameter of ≤ 0.5 cm, were classified as the early‐stage group or grade 1. In the severe‐stage EMN group, horses have one or multiple nodules with a diameter greater than 0.5 cm. This severe‐stage was also graded into 2 to 4, depending on its lesion severity, which was considered extensive confluent melanoma, exophytic tumours with wet surfaces and ulceration, and metastasis in different organs.

**TABLE 1:** The grey horses' signalments, group classification, EMN stage and sampling location.

ID	Age (years)	Sex	Breed	Weight (kg)	EMN stage^a^	Sampling location
Grey horses with the normal‐stage group (*n* = 6)						
NR1	13	Mare	Mixed bred	438	0	Ventral tail base
NR2	7	Mare	Mixed bred	432	0	Ventral tail base
NR3	4	Gelding	Mixed bred	330	0	Ventral tail base
NR4	6	Gelding	Arabian	366	0	Ventral tail base
NR5	4	Mare	Arabian	506	0	Ventral tail base
NR6	4	Mare	Arabian	506	0	Ventral tail base
Grey horses with the early‐stage EMN group (*n* = 7)						
EM1	13	Mare	Mixed bred	532	1	Ventral tail base
EM2	13	Mare	Mixed bred	464	1	Ventral tail base
EM3	15	Gelding	Australian Warmblood	646	1	Ventral tail base
EM4	15	Gelding	Australian Warmblood	625	1	Ventral tail base
EM5	14	Gelding	Australian Warmblood	578	1	Ventral tail base
EM6	15	Mare	Pony	275	1	Ventral tail base
EM7	8	Gelding	Pony	260	1	Ventral tail base
Grey horses with the severe‐stage EMN group (*n* = 6)						
SM1	15	Mare	Mixed bred	511	2	Ventral tail base
SM2	13	Mare	Mixed bred	458	2	Ventral tail base
SM3	14	Gelding	Australian Warmblood	580	3	Ventral tail base
SM4	14	Mare	Australian Warmblood	450	3	Ventral tail base
SM5	15	Gelding	Australian Warmblood	584	2	Ventral tail base
SM6	14	Gelding	Australian Warmblood	570	2	Ventral tail base

^a^ The EMN lesion severity was classified using a modified Desser and colleagues' scoring system: Grade 0, no melanoma; Grade 1, single nodule ≤ 0.5 cm; Grade 2, multiple nodules 0.5–2 cm; Grade 3, nodules ≥ 5 cm; Grade 4, confluent melanoma with skin destruction/metastasis; Grade 5, exophytic metastatic growth [10].

### 2.3 Sample Collection and Protein Extraction

Tissue biopsy samples (*n* = 19) were collected from the ventral tail base of grey horses. Approximately 100 mg of frozen tissue per sample was homogenised in liquid nitrogen using a mortar and pestle. The resulting homogenate was dissolved by 0.1% sodium dodecyl sulfate (SDS), then centrifuged at 10 000 × g for 10 min. The supernatant was collected for further analysis. Total protein isolated from cells was measured with Lowry assay using bovine serum albumin as a standard [11]. Protein concentration of each sample was adjusted to a final concentration of 10 μg/μL and subjected to phosphoprotein enrichment procedure (Phosphoprotein Enrichment Kit, Pierce, IL, USA). The enriched phosphoprotein was concentrated using a 9 kDa cut‐off membrane column (Phosphoprotein Enrichment Kit, Pierce, IL, USA) and desalted by gel filtration (Thermo Scientific, Rockford, IL, USA).

### 2.4 Protein Digestion for LC–MS/MS Analysis

For in‐solution digestion, 5 μg of total protein was dissolved in 10 mM ammonium bicarbonate (AMBIC). Proteins were reduced by 5 mM dithiothreitol (DTT) at 60°C for 1 h and alkylated with 15 mM iodoacetamide (IAA) in the dark at room temperature for 60 min. Digestion was performed by adding sequencing‐grade trypsin (1:20 enzyme‐to‐substrate ratio, Promega, Germany), followed by overnight incubation at 37°C. The resulting peptides were dried and reconstituted in 0.1% formic acid prior to LC–MS/MS analysis.

### 2.5 Liquid Chromatography–Tandem Mass Spectrometry (LC/MS–MS)

Tryptic peptide samples were analysed using an Ultimate3000 Nano/Capillary LC System (Thermo Scientific, UK) coupled to a ZenoTOF 7600 mass spectrometer (SCIEX, Framingham, MA, USA) equipped with a Nano‐captive spray ion source. For each analysis, 100 ng of digested peptides were loaded onto a C18 Pepmap 100 precolumn (300 μm i.d. × 5 mm, 5 μm, 100 Å; Thermo Scientific) and separated on an Acclaim PepMap RSLC C18 analytical column (75 μm i.d. × 15 cm, 2 μm, 100 Å, nanoViper; Thermo Scientific), as well as maintained at 60°C. Chromatographic separation was performed using a 30‐min linear gradient of 5%–55% solvent B (0.1% formic acid in 80% acetonitrile) in solvent A (0.1% formic acid in water) at a constant flow rate of 0.30 μL/min.

The ZenoTOF 7600 mass spectrometer was operated with the following source and gas parameters: ion source gas 1, 8 psi; curtain gas, 35 psi; CAD gas, 7 psi; source temperature, 200°C; positive polarity; and spray voltage, 3300 V. Data‐dependent acquisition (DDA) was performed by selecting the top 50 most abundant precursor ions (intensity threshold > 150 cps) from each survey MS1 scan for MS/MS analysis. Precursor ions were dynamically excluded for 12 s after two MS/MS events, with dynamic collision energy enabled. MS2 spectra were collected from 100 to 1800 m/z with a 50‐ms accumulation time, and the Zeno trap was enabled. Collision energy parameters included a declustering potential of 80 V, with no optimising declustering potential (DP) or collision energy (CE) spread. Time bins were summed (all channels enabled) using a 150 000 cps Zeno trap threshold. The cycle time for the Top 60 DDA method was 3 s. Each sample was analysed in triplicate.

### 2.6 Quantification and Identification of Phosphoproteins

The protein quantity in individual samples was determined using MaxQuant 2.5.0.0 with the Andromeda search engine [12]. Label‐free quantification was performed using MaxQuant's standard settings. Key parameters included a maximum of two missed cleavages, a mass tolerance of 0.6 Da for the main search, and trypsin as the digesting enzyme. Carbamidomethylation of cysteine was set as a fixed modification. Variable modifications included oxidation of methionine, acetylation of the protein N‐terminus, and phosphorylation of serine, threonine, and tyrosine residues. For protein identification, only peptides with a minimum of seven amino acids and at least one unique peptide was considered. Identified proteins require at least two peptides, including one unique peptide, for further data analysis. The protein FDR was set at 1%, estimated using reverse search sequences. The maximal number of modifications per peptide was set to 5. The *Equus caballus* proteome, which was downloaded from UniProt on 11 March 2024 [13], was used in FASTA format. The FASTA file provided by MaxQuant was automatically added to the search space by the software. The raw MS/MS spectra data is available in ProteomeXchange: JPST003894 and PXD065534.

### 2.7 Differentially Expressed Phosphoproteins (DEPhs) Analysis and Functional Pathway Annotation

The DEPhs analysis was conducted across normal‐stage, early‐stage, and severe‐stage EMN using expression levels of each phosphoprotein. The significance of DEPhs was assessed using the Wald test in DESeq2, with log2 fold changes (log2FC) estimated via the lfcShrink function applying the ‘ashr’ adaptive shrinkage method. Multiple testing correction was performed using the Benjamini‐Hochberg (BH) procedure to calculate the false discovery rate (FDR), with significant phosphoproteins identified at FDR < 0.05 [14]. Significant phosphoproteins were identified as potential candidates associated with EMN severity and molecular alterations. Annotation of these DEPhs was achieved through the KEGG database, providing KEGG Orthology (KO) IDs. The obtained KO IDs were categorised into biological categories and specific pathways [15].

**FIGURE 1:**
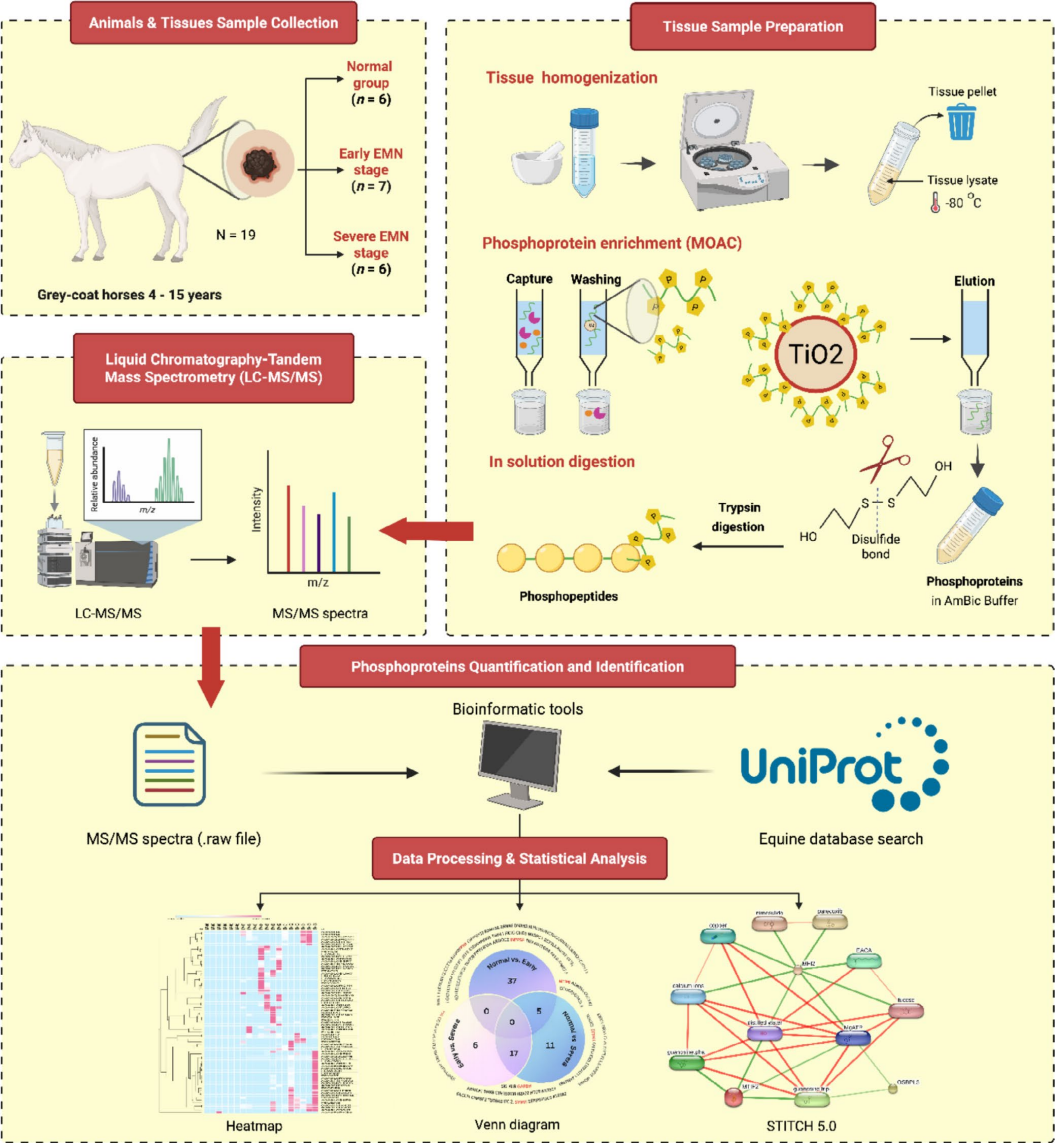
Tissue samples phosphoproteomic workflow characterisation and differentially expressed phosphoproteins (DEPhs) in EMN tissue samples. Created in BioRender. Chotikaprakal, T. (2026) https://BioRender.com/2md96pj.

To visualise uniquely and co‐expressed DEPhs between normal vs. early (N vs. E), normal vs. severe (N vs. S), and early vs. severe (E vs. S) tissue biopsies, a Venn diagram was generated using jvenn (http://jvenn.toulouse.inra.fr/) [16]. To infer potential protein interactions and regulatory mechanisms influenced by phosphorylation, the STITCH database (www.stitch.embl.de) was employed [17]. Specifically, a significant altered phosphoprotein was identified under adjusted *p*‐value < 0.05 in tissue biopsy samples to further construct protein–protein interaction (PPI) networks. STITCH predicted these phosphoprotein‐protein‐chemical interactions in *Equus caballus* by computational predictions between organisms [17]. The overall phosphoproteomic profiling workflow, including DEPhs analysis and KEGG annotation, is shown in Figure 1.

## 3 Results

In accordance with the EMN lesion scoring system developed by Desser and colleagues, a total of 19 grey horses were subjected to classification [10]. The animals were further categorised based on the severity of their EMN lesions, which were classified as normal‐stage (grade 0), early‐stage EMN (grade 1) and severe‐stage EMN (grades 2–4), considering the location of the EMN lesions and signalments. The results provide a clinical classification of the EMN category in grey horses as shown in Table 1. In our study, early‐stage and severe‐stage EMN lesions were primarily found in mature horses, with both groups having a median age of 14 years, which is considerably older than the 5‐year median age for the normal‐stage group.

Our study analysed phosphoprotein data from 19 samples of grey horses, resulting in the identification of 2085 phosphoproteins in tissue biopsy samples. To measure phosphoprotein levels, we used individual mass spectral counts, yielding spectral counts from tissue biopsy samples. A total of 2085 phosphoproteins were identified across all tissue biopsy samples, comprising 2038 annotated and 47 unannotated phosphoproteins. The number of annotated phosphoproteins was highest in the normal‐stage group, with 2059 spectral counts, followed by the early‐stage group, with 1485 spectral counts, and the severe‐stage group, with 673 spectral counts. The details of phosphoproteomic profiling and total spectral counts in tissue biopsies are shown in Table 2.

**TABLE 2:** A comparative analysis of phosphoproteomic profiling and total spectral counts in tissue biopsies.

Phosphoprotein annotation	Tissue biopsy			
	Number of phosphoproteins	Total spectral counts (spectral counts per sample)		
		Normal‐stage	Early‐stage EMN	Severe‐stage EMN
Annotated phosphoproteins	2038	2059	1485	673
Unannotated phosphoproteins	47	10 451	13 110	11 837
Total	2085	12 510	14 595	12 510

### 3.1 Analysis of Differentially Expressed Phosphoproteins and Functional Annotation of Grey Horses Across EMN Stages

**FIGURE 2:**
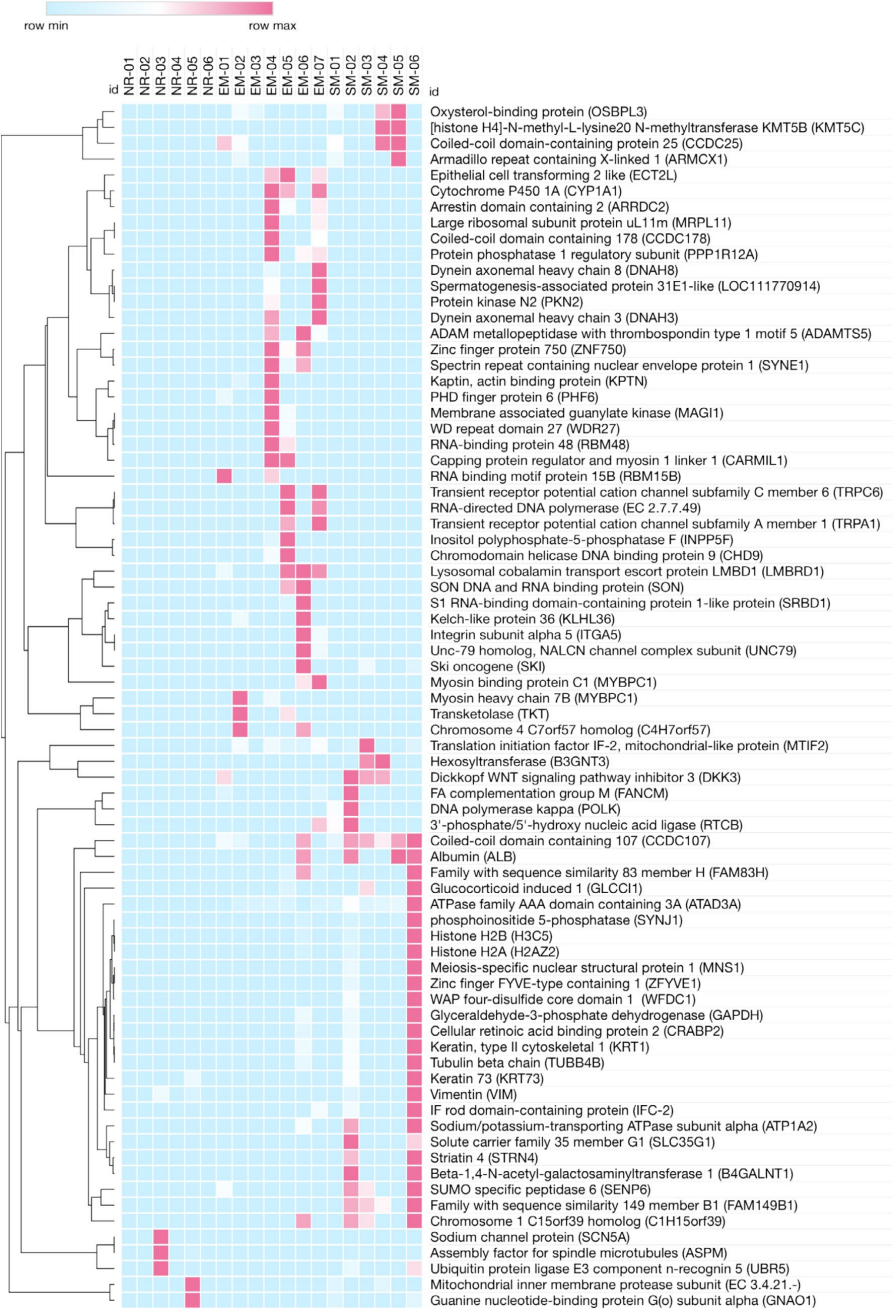
Heatmaps represent the comparison of tissue biopsy phosphoprotein expression levels in response to stage‐specific EMN. This heatmap is generated from the intensity of significant phosphoproteins in the database for each group using the CLUE program (https://clue.io/command, accessed on 10 February 2026), for conception. N, E and S represent normal, early and severe, respectively.

DEPhs analysis of tissue biopsies identified 2085 annotated phosphoproteins. Intensities were normalised within and across samples, relative to each horse, to account for inter‐individual variability. Initial DEPhs analysis using DESeq2 revealed 1825 significant phosphoproteins in N vs. E, 1722 in N vs. S, and 1097 significant phosphoproteins in E vs. S groups when was considered a *p*‐value < 0.05. After adjusting *p*‐value < 0.05, 42 significant phosphoproteins in the N vs. E, 33 in the N vs. S and 17 significant phosphoproteins in the E vs. S groups were identified with full lists provided in Supporting File S2. Overall phosphoprotein expression profiling across normal‐stage, early‐stage EMN and severe‐stage EMN groups are visualised in Figure 2.

**FIGURE 3:**
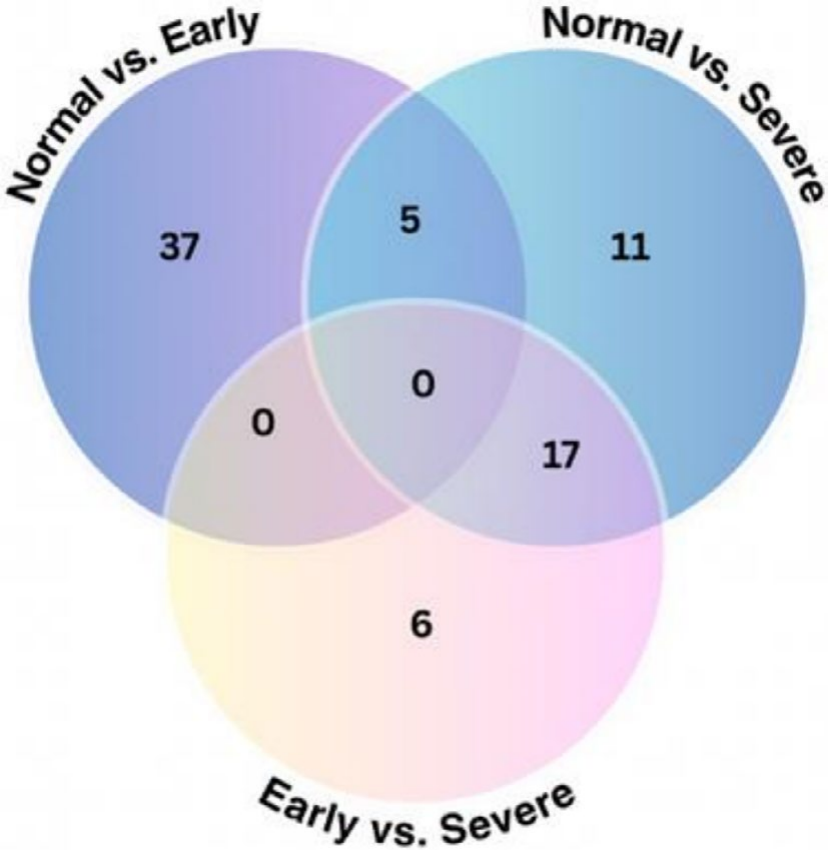
Venn diagram of differentially expressed phosphoproteins in tissue biopsy samples is illustrated using the method described by Bardou and colleagues [16] (http://jvenn.toulouse.inra.fr/) accessed on 1 February 2026. Generated by Canvas.

Venn diagram analysis was performed to identify unique phosphoproteins across the compared groups (Figure 3). This approach provided a comprehensive overview of phosphoprotein expression patterns, revealing 37 unique phosphoproteins in the comparison of N vs. E tissue biopsies. Additionally, 11 unique phosphoproteins were identified in the N vs. S comparison. Notably, 5 phosphoproteins were common to both the N vs. E and N vs. S groups. Furthermore, 17 phosphoproteins were identified between the N vs. S and E vs. S. In addition, 6 phosphoproteins were uniquely identified in the E vs. S comparison. A detailed list of significant phosphoproteins and their associated functions across EMN stages is presented in Table 3.

**TABLE 3:** List of significant phosphoproteins (*p* < 0.01) and associated functions across EMN stages follows KEGG categories.

Protein ID	Protein function	Specific biological pathways/molecular function	*p* < 0.01
37 Significant phosphoproteins in N vs. E group			
A0A3Q2HED2	Epithelial cell transforming 2 like (ECT2L)	Guanyl‐nucleotide exchange factor activity^a^	< 0.01
A0A9L0SG76	Family with sequence similarity 83 member H (FAM83H)	Protein kinase binding^a^	< 0.01
F7AMG5	Kaptin, Actin binding protein (KPTN)	Actin binding^a^	< 0.01
A0A9L0TJF0	Lysosomal cobalamin transport escort protein LMBD1 (LMBRD1)	Vitamin digestion and absorption	< 0.01
A0A9L0TMD7	Cytochrome P450 1A (CYP1A1)	Steroid hormone biosynthesis	< 0.01
A0A9L0SPC8	FA complementation group M (FANCM)	Fanconi anaemia pathway	< 0.01
F7AQF6	Large ribosomal subunit protein uL11m (MRPL11)	Ribosome	< 0.01
K9KB23	S1 RNA‐binding domain‐containing protein 1‐like protein (SRBD1)	Nucleic acid binding^a^	< 0.01
A0A3Q2LFZ9	Coiled‐coil domain containing 178 (CCDC178)	Protein binding^a^	< 0.01
F7ACI3	ADAM metallopeptidase with thrombospondin type 1 motif 5 (ADAMTS5)	Hydrolase and protease^a^	< 0.01
A0A9L0SUH7	Zinc finger protein 750 (ZNF750)	Activator^a^	< 0.01
A0A3Q2HGY6	Transient receptor potential cation channel subfamily C member 6 (TRPC6)	cGMP‐PKG signalling pathway	< 0.01
A0A9L0T0G0	Protein phosphatase 1 regulatory subunit (PPP1R12A)	cAMP signalling pathway	< 0.01
F6YID5	Arrestin domain containing 2 (ARRDC2)	Protein binding and protein transport^a^	< 0.01
F6STH6	Inositol polyphosphate‐5‐phosphatase F (INPP5F)	Inositol phosphate metabolism (phosphatase activity)	< 0.01
A0A9L0SSP7	Dynein axonemal heavy chain 8 (DNAH8)	Motor proteins	< 0.01
F6QLX7	Integrin subunit alpha 5 (ITGA5)	Virion—Herpesvirus	< 0.01
F6ZMK0	PHD finger protein 6 (PHF6)	ATP‐dependent chromatin remodelling	< 0.01
A0A5F5PSW9	Spermatogenesis‐associated protein 31E1‐like (LOC111770914)	Spermatogenesis and cell differentiation^a^	< 0.01
F6XYA5	Membrane associated guanylate kinase, WW and PDZ domain containing 1 (MAGI1)	Rap1 signalling pathway	< 0.01
A0A9L0SVF8	RNA‐directed DNA polymerase (EC 2.7.7.49)	Reverse Transcriptase activity^a^	< 0.01
F6RHE9	Spectrin repeat containing nuclear envelope protein 1 (SYNE1)	Cytoskeleton in muscle cells	< 0.01
A0A5F5PNA0	SON DNA and RNA binding protein (SON)	RNA and DNA binding^a^	< 0.01
A0A9L0RLC5	Myosin heavy chain 7B (MYH7B)	Motor proteins	< 0.01
A0A9L0RDM7	Transient receptor potential cation channel subfamily A member 1 (TRPA1)	Inflammatory mediator regulation of TRP channels	< 0.01
A0A3Q2HKC5	Protein kinase N2 (PKN2)	PI3K‐Akt signalling pathway	< 0.01
A0A9L0RNI8	Chromodomain helicase DNA binding protein 9 (CHD9)	ATP binding activity & DNA‐dependent ATPase activity^a^	< 0.01
A0A3Q2L3W6	Myosin binding protein C1 (MYBPC1)	Cytoskeleton in muscle cells	< 0.01
A0A9L0RCN4	WD repeat domain 27 (WDR27)	Protein binding^a^	< 0.01
A0A9L0RPM9	Transketolase (TKT)	Pentose phosphate pathway	< 0.01
F7CB19	Chromosome 4 C7orf57 homologue (C4H7orf57)	Uncharacterised protein^a^	< 0.01
A0A3Q2L982	RNA binding motif protein 15B (RBM15B)	RNA and DNA binding^a^	< 0.01
A0A9L0T8T3	RNA‐binding protein 48 (RBM48)	RNA and DNA binding^a^	< 0.01
A0A3Q2HZA5	Dynein axonemal heavy chain 3 (DNAH3)	Motor proteins	< 0.01
A0A9L0SG46	Kelch‐like protein 36 (KLHL36)	Ubl conjugation pathway^a^	< 0.01
F6ZA14	Unc‐79 homologue, NALCN channel complex subunit (UNC79)	Protein binding^a^	< 0.01
A0A9L0STI3	Capping protein regulator and myosin 1 linker 1 (CARMIL1)	Actin capping protein regulation^a^	< 0.01
5 Significant phosphoproteins in N vs. E and N vs. S groups			
A0A9L0S528	Coiled‐coil domain containing 107 (CCDC107)	Protein binding^a^	< 0.01
K9K9R4	Coiled‐coil domain‐containing protein 25 (CCDC25)	DNA binding activity^a^	< 0.01
A0A3Q2HCM1	Oxysterol‐binding protein (OSBPL3)	Lipid binding	< 0.01
K9KB47	Translation initiation factor IF‐2, mitochondrial‐like protein (MTIF2)	GTP binding^a^	< 0.01
A0A3Q2LEH6	ATPase family AAA domain containing 3A (ATAD3A)	ATP hydrolysis activity^a^	< 0.01
17 Significant phosphoproteins in N vs. S group			
A0A9L0SF85	Glucocorticoid induced 1 (GLCCI1)	Regulation of kinase activity^a^	< 0.01
F7AZM6	Phosphoinositide 5‐phosphatase (SYNJ1)	Inositol phosphate metabolism	< 0.01
F6VWI6	SUMO specific peptidase 6 (SENP6)	Protein modification; protein sumoylation^a^	< 0.01
F6WE78	Dickkopf WNT signalling pathway inhibitor 3 (DKK3)	Wnt signalling pathway^a^	< 0.01
A0A9L0TCZ2	Ski oncogene (Proto‐oncogene c‐Ski) (SKI)	TGF‐beta signalling pathway	< 0.01
A0A9L0R2L2	Histone H2B (H3C5)	Neutrophil extracellular trap formation	< 0.01
A0A5F5PEW7	Histone H2A (H2AZ2)	ATP‐dependent chromatin remodelling	< 0.01
F6QDE9	Sodium/potassium‐transporting ATPase subunit alpha (ATP1A2)	cAMP signalling pathway	< 0.01
A0A3Q2HE84	Armadillo repeat containing X‐linked 1 (ARMCX1)	Haematopoietic stem cell homeostasis	< 0.01
F6Y457	Glyceraldehyde‐3‐phosphate dehydrogenase (GAPDH)	Glycolysis/gluconeogenesis	< 0.01
L7MS24	3′‐phosphate/5′‐hydroxy nucleic acid ligase (RTCB)	RNA ligase (GTP) activity^a^	< 0.01
A0A5F5PMV0	Keratin, type II cytoskeletal 1 (KRT1)	Signalling receptor activity^a^	< 0.01
A0A3Q2H3R5	Tubulin beta chain (TUBB4B)	Phagosome	< 0.01
A0A3Q2I854	IF rod domain‐containing protein (IFC‐2)	Oestrogen signalling pathway	< 0.01
A0A9L0RV99	Cellular retinoic acid binding protein 2 (CRABP2)	Lipid binding^a^	< 0.01
F6QEN8	Chromosome 1 C15orf39 homologue (C1H15orf39)	Protein and lipid binding^a^	< 0.01
A0A9L0S6J2	Albumin (ALB)	Thyroid hormone synthesis	< 0.01
11 Significant phosphoproteins in N vs. S and E vs. S groups			
A0A9L0S5Q1	Striatin 4 (STRN4)	MAPK signalling pathway—fly	< 0.01
A0A9L0R112	[histone H4]‐N‐methyl‐L‐lysine20 N‐methyltransferase KMT5B (KMT5C)	Lysine degradation	< 0.01
A0A5F5PNS0	DNA polymerase kappa (POLK)	DNA binding^a^	< 0.01
A0A5F5PKS6	Sodium channel protein (SCN5A)	Adrenergic signalling in cardiomyocytes	< 0.01
A0A9L0TFB6	Solute carrier family 35 member G1 (SLC35G1)	Nucleotide‐sugar transporter^a^	< 0.01
A0A9L0SVX5	Meiosis‐specific nuclear structural protein 1 (MNS1)	Meiotic cell cycle^a^	< 0.01
F6W154	Hexosyltransferase (B3GNT3)	Mucin type O‐glycan biosynthesis	< 0.01
F6WCQ8	Zinc finger FYVE‐type containing 1 (ZFYVE1)	Autophagy—animal	< 0.01
F6SC02	WAP four‐disulfide core domain 1 (WFDC1)	Protease inhibitor activity^a^	< 0.01
A0A3Q2GXU4	Beta‐1,4‐N‐acetyl‐galactosaminyltransferase 1 (B4GALNT1)	Sphingolipid metabolism	< 0.01
A0A9L0S8F9	Family with sequence similarity 149 member B1 (FAM149B1)	Protein binding^a^	< 0.01
6 Significant phosphoproteins in E vs. S group			
A0A9L0TRA7	Vimentin (VIM)	Cytoskeleton in muscle cells	< 0.01
K9K261	Mitochondrial inner membrane protease subunit (EC 3.4.21.‐)	Protein export	< 0.01
F7AGY4	Keratin 73 (KRT73)	Protein binding^a^	< 0.01
A0A9L0T1F9	Guanine nucleotide‐binding protein G(o) subunit alpha (GNAO1)	Rap1 signalling pathway	< 0.01
A0A9L0T4C7	Assembly factor for spindle microtubules (ASPM)	Mitotic spindle regulation^a^	< 0.01
A0A3Q2HKH3	Ubiquitin protein ligase E3 component n‐recognin 5 (UBR5)	Ubiquitin mediated proteolysis	< 0.01

^a^ Stands for a predicted function, while the molecular functions and biological processes themselves were inferred from UniProt [13].

### 3.2 Identification of Significant Phosphoproteins and the Metabolic Functions in Relation to Melanoma Interaction

To identify key regulatory proteins, we focused on phosphoproteins in pathways such as inositol phosphate metabolism, for example, inositol polyphosphate‐5‐phosphatase F (INPP5F), protein kinase N2 (PKN2), phosphoinositide 5‐phosphatase (SYNJ1), and carbohydrate metabolism, for example, transketolase (TKT), glyceraldehyde‐3‐phosphate dehydrogenase (GAPDH).

**FIGURE 4:**
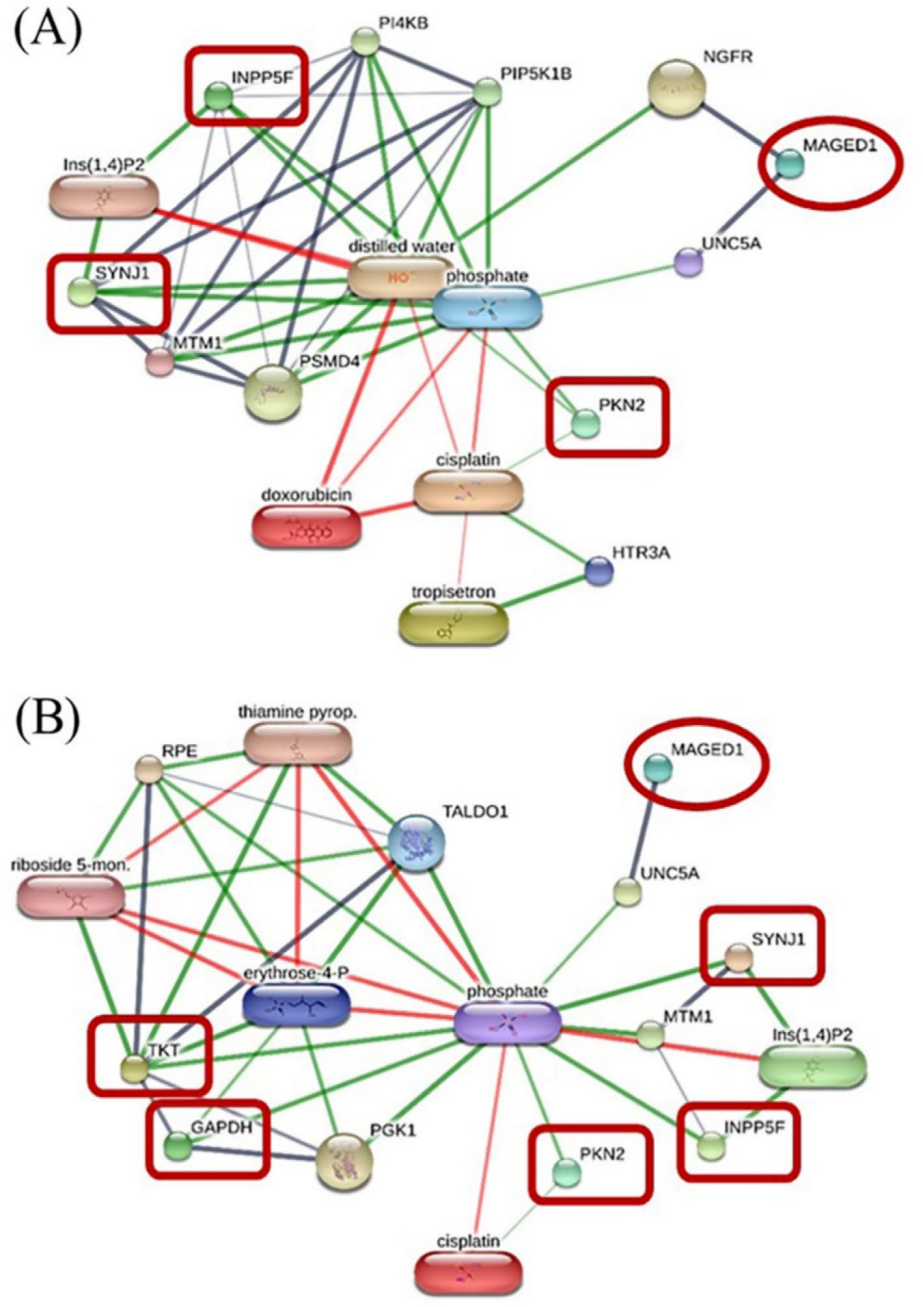
The networks of the comprehensive phosphoprotein expression pattern. (A) melanoma‐associated antigen D1 (MAGED1) with the significant phosphoproteins interaction in INPP5F, PKN2, SYNJ1 interaction with chemotherapy; cisplatin and doxorubicin, antineoplastic cancer; tropisetron. (B) MAGED1 with the significant phosphoproteins interaction in inositol phosphate metabolism and carbohydrate metabolism: INPP5F, PKN2, SYNJ1, TKT, GADPH interaction with chemotherapy; cisplatin in *Equus caballus* that computational prediction between organism and interaction aggregated by the Stitch program [17] (www.stitch.embl.de, accessed on 17 June 2025) Version 5.0.

The comprehensive interaction network for these proteins, computationally predicted for *Equus caballus* using the STITCH database (version 5.0) [17], is shown in Figure 4. The network highlights notable phosphoproteins that interact with the melanoma antigen family.

## 4 Discussion

EMN is a common skin condition in grey horses, typically appearing at 5 years of age or older, with an incidence that progressively increases [18]. While often initially benign and slow‐growing, early lesions are frequently undetected due to their subtle presentation. EMN manifests in three recognised clinical patterns: most commonly, indolent growth without metastasis; less frequently, malignant transformation from benign melanocytomas; and rarely, primary malignancy [19]. Tumours that persist for over 6 years often give rise to multiple lesions. Approximately two‐thirds of affected horses eventually progress to an aggressive, malignant stage, underscoring the critical importance of early detection for improved prognosis [2]. Despite histological similarities, dermal melanoma and dermal melanomatosis diverge significantly in clinical behaviour; the former is surgically manageable, whereas the latter exhibits metastatic potential and poor therapeutic response, supporting a model of stepwise progression [20].

Our study identified EMN at a median age of 14 years, notably earlier than previous estimates (~15 years in 80% of cases). This finding highlights the importance of investigating disease onset in mature rather than solely aged horses. Understanding EMN development in this earlier age group could reveal novel pathogenic drivers or vulnerability factors that are currently overlooked, potentially leading to more effective preventive or early therapeutic interventions. Ultimately, this approach will enable us to uncover molecular mechanisms associated with early lesions, thereby enhancing diagnostic accuracy and refining intervention strategies.

This study employed a phosphoproteomic approach to elucidate the dynamic changes in protein phosphorylation that occur during EMN progression. These changes reveal critical shifts in cell signalling and metabolism, particularly in carbohydrate pathways that fuel tumour growth and survival. Such findings suggest that early molecular changes, driven by specific phosphorylation events, may initiate and sustain EMN progression. Unlike mere protein expression profiling, phosphoproteomics offers a unique window into the real‐time, active regulation of proteins within cells [21]. This makes it an especially valuable tool for cancer research, enabling us to map disrupted signalling networks, identify potential therapeutic targets, and gain a better understanding of treatment resistance mechanisms. For EMN, where early‐stage diagnosis and targeted therapies remain limited, the in‐depth molecular perceptions gained from phosphoproteomics are crucial for developing more precise and timely interventions.

### 4.1 Identification of Differentially Expressed Phosphoproteins Throughout the Early Stage of EMN

Our phosphoproteomic analysis reveals the stage‐specific dysregulation of phosphoinositide metabolism in EMN, with Phosphorylated INPP5F exhibiting significant phosphorylation in early EMN stages. As a phosphatase that typically dephosphorylates PI(3,4,5)P_3_, INPP5F acts as a negative regulator of the canonical phosphoinositide 3‐kinase (PI3K)/protein kinase B (AKT) pathway [22]. Under normal physiological conditions, INPP5F functions as a tumour suppressor by inhibiting the PI3K/AKT signalling pathway through dephosphorylation, thereby maintaining cellular homeostasis and preventing uncontrolled cell growth. However, phosphorylation‐induced post‐translational modifications of INPP5F can disrupt its regulatory role, leading to mislocalisation or loss of substrate specificity [23]. This results in sustained accumulation of Phosphatidylinositol 3,4,5‐trisphosphate (PI(3,4,5)P_3_) and persistent AKT activation, which drives cancer initiation by promoting pre‐malignant cell survival through apoptosis inhibition, inducing somatic cell dedifferentiation into stem‐like states, and enhancing cancer stem cell (CSC) pathways like sex‐determining Region Y‐Box 2 (SOX2), Nanog homeobox (NANOG), and octamer‐binding transcription factor 4 (OCT4) expression [24]. In early‐stage transformed cells (e.g., EMN), hyperphosphorylation of INPP5F releases the brake on PI3K/AKT signalling, establishing a pro‐oncogenic environment before genetic mutations accumulate and initiate CSC‐like properties. Feedback loops between INPP5F and mechanistic target of rapamycin complex 2 (mTORC2) amplify AKT phosphorylation at Serine 473 (Ser473), a hallmark of constitutive AKT activation in CSCs and many cancers [25]. This dysregulation highlights how INPP5F phosphorylation serves as an early trigger for oncogenic transformation, linking metabolic changes to malignant progression [23, 25].

PKN2 was observed to be activated in the early stage of EMN through its increased kinase activity and phosphorylation status, which promotes key signalling events linked to tumour initiation. PKN2 can modulate the PI3K‐Akt‐mTOR signalling pathway, which is crucial for cell survival, proliferation, and metabolic adaptation [26]. PKN2 facilitates the phosphorylation of Akt at threonine 308, a critical activation site, thereby enhancing Akt kinase activity [27]. Activated Akt subsequently phosphorylates downstream targets, including mTOR, which regulates protein synthesis, cell growth, and metabolism. Through this pathway, PKN2 promotes anabolic metabolism and antioxidant responses by stabilising nuclear factor erythroid 2‐related factor 2 (NRF2), increasing nicotinamide adenine dinucleotide phosphate (NADPH) production, and enhancing cellular resistance to oxidative stress [28]. This metabolic reprogramming and enhanced survival signalling enable early stage EMN cells to resist apoptosis and proliferate, driving tumour initiation. Additionally, PKN2's role in cytoskeletal remodelling may facilitate early tumour cell motility and invasion [29]. Thus, PKN2 acts as a key modulator linking extracellular signals to PI3K‐Akt‐mTOR pathway activation, fostering the survival and expansion of initiated melanocytic cells in early‐stage EMN.

TKT, a thiamine diphosphate‐dependent enzyme, plays a critical mechanistic role in the initiation and early stages of melanoma by regulating metabolic reprogramming, redox homeostasis and tumour progression. As a key enzyme in the non‐oxidative branch of the pentose phosphate pathway (PPP), TKT facilitates the production of ribose‐5‐phosphate (R5P), essential for nucleotide synthesis and NADPH, which maintains cellular redox balance by reducing reactive oxygen species (ROS) [30]. This metabolic support is crucial for rapidly proliferating melanoma cells during the initiation of tumours. In melanoma, aberrant expression of transketolase‐like 1 (TKTL1), a homologue of TKT, is frequently observed due to DNA hypomethylation of its promoter, leading to its ectopic expression [31]. TKTL1 promotes the Warburg effect‐enhanced aerobic glycolysis, even in the presence of oxygen, providing energy and biosynthetic precursors necessary for early tumour growth and invasion [30]. Functional studies have demonstrated that TKTL1 enhances glucose consumption, lactate production, cell proliferation, and invasive capabilities of melanoma cells, linking metabolic reprogramming directly to tumour aggressiveness [31]. Moreover, TKT has influenced multiple oncogenic signalling pathways, including mTOR, PI3K/AKT and MAPK, which are pivotal in melanoma initiation and progression [32]. Its role in modulating epithelial‐mesenchymal transition (EMT) through downregulation of E‐cadherin further facilitates early melanoma cell migration and invasion [33]. Additionally, TKT contributes to immune evasion by upregulating immune checkpoint molecules, such as programmed death‐ligand 1 (PD‐L1), through the ROS‐mTOR axis, thereby creating a microenvironment conducive to tumour survival [32]. The present study revealed that TKT is highly expressed in early‐stage melanoma samples. Due to its diverse functions, TKT holds great potential as both a biomarker and a therapeutic target for early melanoma intervention.

Altogether, the dysregulation or upregulation of these proteins that is, phosphorylated INPP5F, PKN2, and TKT, not only disrupts normal cellular signalling and metabolic homeostasis but also collectively fosters a microenvironment conducive to melanoma initiation. Their stage‐specific alterations highlight their potential as early biomarkers and therapeutic targets for intercepting melanoma at its inception.

### 4.2 Identification of Differentially Expressed Phosphoproteins Throughout the Severe Stage of EMN

SYNJ1 plays a significant role in melanoma progression, with its phosphorylation markedly increased in severe stages of the EMN. SYNJ1 is an enzyme involved in phosphoinositide metabolism, which regulates key cellular processes, including membrane trafficking, signal transduction and cytoskeletal dynamics. In melanoma, the enhanced phosphorylation of SYNJ1 likely alters its enzymatic activity or localisation, contributing to the dysregulation of phosphoinositide signalling pathways that promote tumour progression and metastasis. Although detailed mechanistic studies specifically on SYNJ1 phosphorylation in melanoma are limited, its role in phosphoinositide regulation suggests that aberrant SYNJ1 activity could enhance oncogenic signalling cascades such as PI3K/AKT and mitogen‐activated protein kinase (MAPK) pathways, which are well‐established drivers of melanoma progression and metastasis [34]. Therefore, SYNJ1 phosphorylation in severe melanoma stages may represent a critical molecular event that supports aggressive tumour behaviour and could serve as a potential biomarker or therapeutic target in advanced melanoma.

GAPDH plays a crucial role in severe melanoma stages primarily through its involvement in cancer cell metabolism and survival [35]. Melanoma cells exhibit a high aerobic glycolytic rate (the Warburg effect), relying heavily on glycolysis for ATP production and biosynthesis [36]. GAPDH, a key glycolytic enzyme, is essential for this metabolic adaptation. Studies have shown that inhibition or oxidation of GAPDH activity in melanoma cells leads to a significant reduction in glycolysis, as evidenced by decreased lactate production, and results in reduced melanoma cell viability, while normal melanocytes are less affected. This indicates that melanoma cells are more dependent on GAPDH‐mediated glycolysis for their survival compared to normal cells [37]. Beyond its metabolic role, GAPDH also participates in non‐glycolytic functions that contribute to tumour progression, including the regulation of autophagy, DNA repair, and cell cycle progression [35, 38]. It can translocate to the nucleus to induce autophagy‐related proteins and stabilise the mRNAs of cytokines involved in tumour progression, such as colony‐stimulating factor 1 (CSF‐1). GAPDH also interacts with DNA repair enzymes to maintain genomic stability under oxidative stress, a critical process in the harsh tumour microenvironment [38]. These multifunctional roles help melanoma cells resist cell death and sustain proliferation during advanced stages. In summary, GAPDH supports melanoma progression in severe stages by sustaining enhanced glycolysis and contributing to cellular processes that promote survival, proliferation, and resistance to stress. Targeting GAPDH disrupts these processes, highlighting its potential as a biomarker and therapeutic target in advanced melanoma.

The Ski oncogene (Proto‐oncogene c‐Ski; SKI) protein plays a critical oncogenic role in severe melanoma by repressing the tumour‐suppressive effects of the transforming growth factor beta (TGF‐β) signalling pathway. SKI is upregulated progressively during melanoma development, with higher expression correlating with advanced disease stages. It inhibits TGF‐β‐mediated growth inhibition by associating with Smad transcription factors, preventing their nuclear localisation, and promoting phosphorylation of the Smad3 linker region. This modification alters Smad3's function from tumour suppression to oncogenesis, resulting in increased expression of oncogenic proteins such as cellular myelocytomatosis oncogene (c‐MYC) and plasminogen activator inhibitor‐1 (PAI‐1), which promote tumour growth, angiogenesis, and invasion [39]. Moreover, SKI enhances the proliferation, migration, and survival of melanoma cells by modulating multiple pathways. It promotes the expression of microphthalmia‐associated transcription factor (MITF) and neuronal cell adhesion molecule (Nr‐CAM), proteins involved in melanoma cell survival and motility [40]. SKI's localisation shifts from predominantly nuclear in normal melanocytes and early melanomas to both nuclear and cytoplasmic or exclusively cytoplasmic in advanced and metastatic melanomas, where it continues to inhibit TGF‐β signalling by sequestering Smad proteins in the cytoplasm. Knockdown of SKI in melanoma cells restores TGF‐β tumour suppressor activity, reduces anchorage‐independent growth, and significantly impairs tumour formation in vivo, demonstrating its essential role in melanoma progression [41]. Therefore, SKI acts as a molecular switch that blocks TGF‐β's growth‐inhibitory effects and promotes oncogenic signalling, contributing to melanoma aggressiveness, invasion, and metastasis in severe stages.

Striatin‐4 (STRN4) is a scaffolding protein involved in multiple signalling pathways that regulate cell cycle progression, apoptosis, and cytoskeletal dynamics [42]. Functionally, STRN4 is a scaffolding protein that facilitates the assembly of multiprotein complexes involved in signal transduction. Although extensively studied in cancers such as colon, liver, and prostate cancer, its role in melanoma is emerging, with evidence suggesting that abnormal STRN4 expression contributes to tumour aggressiveness and poor prognosis [43]. In severe stages of melanoma, STRN4 is often overexpressed and mislocalised from the cell membrane to the cytoplasm, correlating with increased tumour size, depth of invasion, higher pathological grade and microvascular invasion [44]. These changes promote melanoma cell proliferation, invasion, and metastasis by activating downstream effectors including RhoA, misshapen‐like kinase 1 and platelet‐derived growth factor receptor‐α (PDGFRA), which facilitate cytoskeletal remodelling and enhanced motility. STRN4's scaffolding function also regulates protein phosphatase 2A (PP2A) complexes, influencing multiple oncogenic signalling cascades that drive melanoma progression [43]. Simultaneously, STRN4 links to enhanced MAPK pathway activity, which drives cytoskeletal remodelling, cell proliferation and invasiveness, hallmarks of malignant transformation [42]. Its elevated expression is associated with worse overall survival, making STRN4 a potential independent prognostic biomarker and therapeutic target in advanced melanoma.

In severe EMN, increased expression of Dickkopf‐3 (DKK3) contributes to tumour progression by modulating the Wnt signalling pathway and shaping a pro‐tumour microenvironment. Although traditionally considered a Wnt antagonist, DKK3 can paradoxically enhance β‐catenin and Yes‐associated protein (YAP)/transcriptional co‐activator with PDZ‐binding motif (TAZ) signalling in this context, promoting melanocyte proliferation, extracellular matrix remodelling, and tumour invasiveness [45]. Additionally, DKK3 supports tumour growth by activating Nuclear Factor kappa‐light‐chain‐enhancer of activated B cells (NF‐κB) pathways and fostering an immunosuppressive environment through the suppression of CD8+ T cell infiltration and the promotion of M2 macrophages [46]. These combined effects facilitate melanocytic transformation, immune evasion, and metastasis, positioning DKK3 as a key regulator and potential therapeutic target in severe EMN.

This upregulation of glucocorticoid‐induced 1 (GLCCI1) observed in severe EMN is mechanistically relevant, as GLCCI1 is known to act downstream of glucocorticoid receptor (GR) signalling, promoting anti‐apoptotic pathways and enhancing tumour cell survival. In the context of EMN, increased GLCCI1 likely contributes to the aggressive phenotype of advanced tumours by supporting GR‐mediated transcription of survival genes for example, B‐cell lymphoma 2 (BCL2) and myeloid cell leukaemia 1 (MCL1), suppressing apoptosis, and facilitating adaptation to cellular stress. Furthermore, pathway analysis in the study linked GLCCI1 to metabolic reprogramming, particularly the PI3K‐Akt and hypoxia‐inducible factor 1 (HIF‐1) signalling pathways, which are crucial for tumour growth under hypoxic and nutrient‐limited conditions [47]. Clinically, high GLCCI1 expression in severe EMN correlated with larger, ulcerated, and metastatic lesions, suggesting its role in tumour progression and poor prognosis. These findings underscore GLCCI1 as both a biomarker of advanced EMN and a potential therapeutic target for interventions aimed at disrupting glucocorticoid‐driven tumour survival mechanisms.

In summary, the dysregulation or altered expression of SYNJ1, GAPDH, SKI protein, Striatin‐4, DKK3 and GLCCI1 converge to promote melanoma progression in severe stages by enhancing oncogenic signalling, metabolic adaptation, immune evasion and tumour cell invasiveness. These proteins serve as promising diagnostic biomarkers for disease severity and as therapeutic targets to disrupt melanoma growth, metastasis and treatment resistance.

### 4.3 Identification of Differentially Expressed Phosphoproteins Throughout the Early and Severe Stage of EMN

Phosphorylation of Mitochondrial Translational Initiation Factor 2 (MTIF2) plays a mechanistic role in both the early and severe stages of melanoma, influencing mitochondrial function and metabolic plasticity [48]. MTIF2 is essential for mitochondrial protein synthesis, which supports oxidative phosphorylation (OXPHOS), a key energy‐producing process in melanoma cells [49]. Although melanoma cells exhibit the Warburg effect with increased glycolysis, recent evidence highlights that OXPHOS remains active and critical, especially through metabolic plasticity where melanoma cells dynamically switch between glycolysis and OXPHOS to adapt to environmental stresses and support tumour progression. The upregulation of mitochondrial pathways, including the translation machinery and OXPHOS components, correlates with increased tumour cell proliferation, survival and chemoresistance in melanoma [50]. Moreover, mitochondrial dynamics, such as fission and fusion, are altered in melanoma, supporting tumour growth and adaptation, processes in which MTIF2 function is implicated [51]. In severe melanoma stages, enhanced MTIF2 phosphorylation may contribute to maintaining mitochondrial integrity and function despite mitochondrial mutations, supporting metabolic symbiosis between glycolytic and oxidative tumour cell populations. This metabolic flexibility confers a survival advantage, promotes invasion and metastasis and contributes to therapy resistance.

### 4.4 Dissecting Protein Phosphorylation Patterns and Associated Pathways Across EMN Progression and Pathogenesis

Vimentin (VIM) upregulation in severe EMN provides molecular evidence for active EMT, enabling tumour dissemination [52]. Together with upregulated SYNJ1 and STRN4, these findings reveal coordinated cytoskeletal remodelling driving the invasive phenotype. Vimentin's conservation as a progression marker across human, canine and equine melanomas underscores EMN's validity as a comparative model. Assembly factor for spindle microtubules (ASPM) upregulation indicates heightened proliferative activity in severe EMN. ASPM's essential role in mitotic spindle assembly and its overexpression in multiple human malignancies correlate with genomic instability and poor outcomes [53]. ASPM represents both a proliferation biomarker and actionable therapeutic target [54]. Guanine nucleotide‐binding protein G(o) subunit alpha (GNAO1) dysregulation implicates Ras‐related protein 1 (Rap1) pathway alterations intersecting with PI3K‐Akt, MAPK and Wnt/β‐catenin signalling [55]. This multi‐pathway convergence creates a permissive environment for progression and suggests combination therapies targeting multiple nodes may be required [56]. This complexity mirrors human melanoma resistance mechanisms. Ubiquitin protein ligase E3 component n‐recognin 5 (UBR5) dysregulation indicates compromised protein quality control [57]. UBR5's regulation of cell cycle, DNA damage response, and signal transduction proteins including Wnt/β‐catenin components may connect to elevated DKK3 observed in severe EMN [58]. Disrupted protein homeostasis may contribute to therapeutic resistance. Mitochondrial protease dysregulation complements upregulated TKT and GAPDH, revealing comprehensive metabolic rewiring [59]. This shifts toward aerobic glycolysis (Warburg effect) and enhanced biosynthetic capacity supports rapid tumour growth and represents potential therapeutic vulnerabilities. Keratin 73 (KRT73) differential expression reflects dedifferentiation during progression [60]. This cellular plasticity contributes to tumour heterogeneity, therapeutic resistance, and metastatic potential, paralleling dedifferentiation processes in human melanoma [61].

Early‐stage EMN features INPP5F dysregulation and PKN2/PI3K‐Akt activation. Progression to severe stage involves: [1] EMT activation (VIM, SYNJ1, STRN4), [2] enhanced proliferation (ASPM), [3] multi‐pathway dysregulation (GNAO1/Rap1, PI3K‐Akt, MAPK, Wnt), [4] metabolic reprogramming (TKT, GAPDH, mitochondrial dysfunction), [5] protein homeostasis failure (UBR5) and [6] dedifferentiation (KRT73). This multifaceted transformation drives aggressive disease. Stage‐specific signatures suggest targeted strategies: early‐stage PI3K‐Akt and inositol phosphate pathway inhibition; severe‐stage combination approaches including anti‐EMT therapies, mitotic inhibitors, multi‐kinase inhibitors, metabolic inhibitors and proteasome inhibitors. VIM and ASPM emerge as top biomarker candidates. Molecular similarities between EMN and human melanoma support bidirectional translational research [62].

**FIGURE 5:**
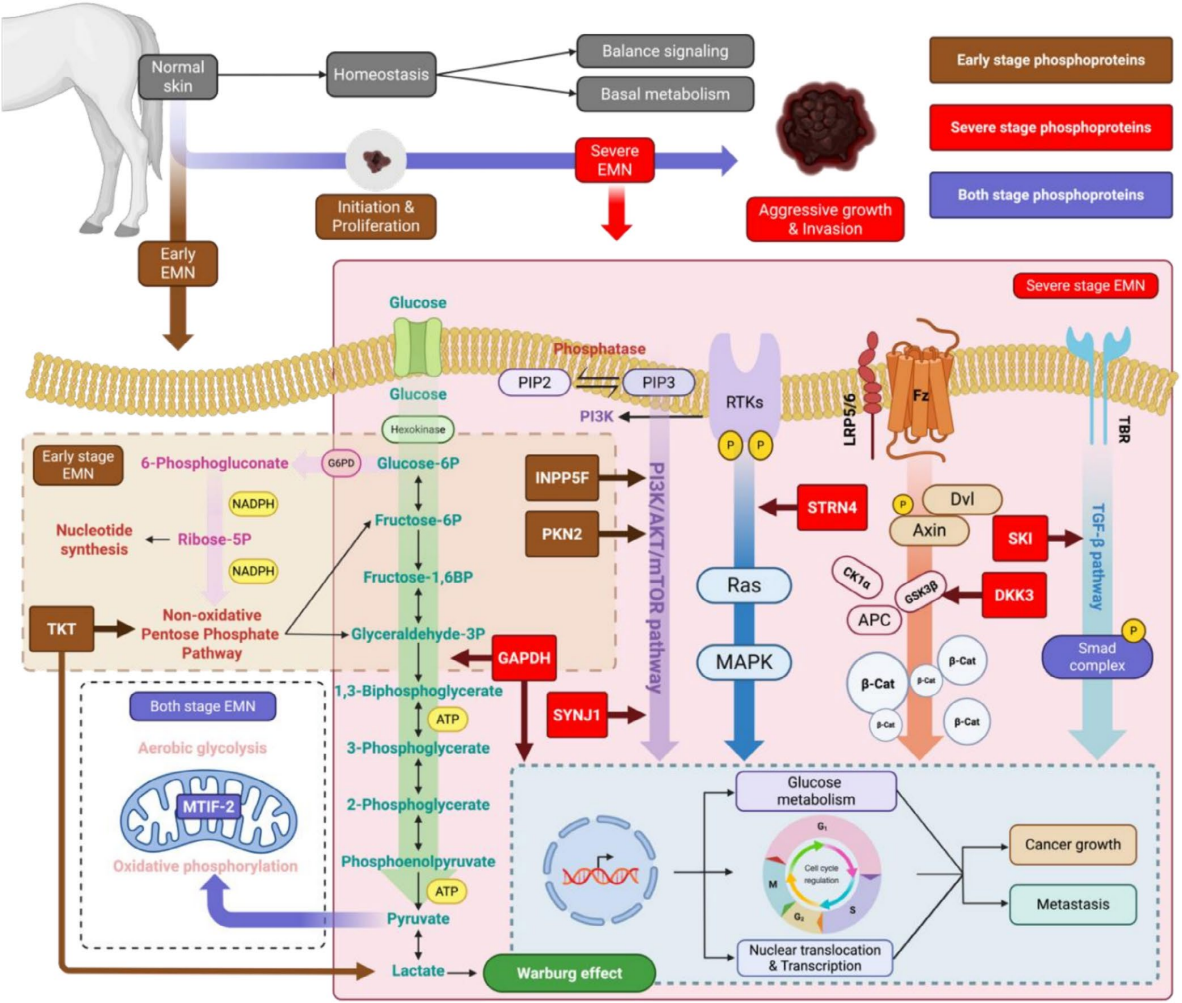
A schematic illustration summarising the roles of phosphoinositide, glycolysis, and the pentose phosphate pathway in carbohydrate metabolism, as well as the Wnt and MAPK functional pathways in EMN pathogenesis, based on tissue biopsied samples. Created in BioRender Tesena, P. (2025) https://BioRender.com/2yfsr3d.

The present study elucidates the pathogenesis of EMN through a series of molecular and cellular changes, reflected in distinct protein phosphorylation patterns at each disease stage (Figure 5). In the normal stage, melanocytes exhibit homeostatic signalling and metabolic activity, with no evidence of neoplastic transformation or abnormal proliferation. In the early stage of EMN, phosphoproteomic profiling reveals a shift marked by the dysregulation of inositol phosphate metabolism and activation of the PI3K‐Akt pathway, primarily driven by decreased phosphorylation of INPP5F and increased activation of PKN2, which together promote melanocyte survival, proliferation and resistance to apoptosis. Concurrently, metabolic reprogramming is evident, with the upregulation of TKT and GAPDH, which enhances glycolysis and the pentose phosphate pathway to supply energy and biosynthetic precursors for rapidly dividing tumour cells—a hallmark of the Warburg effect. The early stage also features the suppression of DKK3, a Wnt pathway antagonist, resulting in depression of Wnt/β‐catenin signalling, which increases tumour cell plasticity and supports neoplastic transformation. As EMN progresses to the severe stage, the tumour microenvironment is further remodelled by the hyperphosphorylation of SYNJ1, which alters membrane trafficking and endocytosis, and by the overexpression and mislocalisation of STRN4, which activates the MAPK pathway and drives cytoskeletal reorganisation, thereby facilitating tumour invasiveness and metastatic potential. Further investigation can be performed for standardisation, staging systems and horse‐specific histopathological criteria for equine melanoma. An experimental validation by Western blotting and phospho‐Immunohistochemistry can be additionally integrated together with the exploration of the real‐time assessment of phosphorylation dynamics in equine melanoma tissues as well as companion diagnostics in tandem with therapeutic agents. Throughout this study, these stage‐specific protein alterations not only drive the progression from benign melanocytic proliferation to aggressive, invasive melanoma, but also highlight potential biomarkers and therapeutic targets for early detection and intervention in EMN.

## 5 Conclusion

This study provides the integrative phosphoproteomic profiling of EMN tissue biopsies, revealing how protein phosphorylation shifts across disease‐specific stages. We identified key phosphoproteins whose distinct regulation defines early stage EMN (involving inositol phosphate metabolism, PI3K‐Akt activation, and metabolic reprogramming) and severe stage EMN (featuring altered membrane/cytoskeletal dynamics and persistent Wnt/β‐catenin and MAPK pathway activation). These disease‐specific stages in relation to phosphoprotein signatures and pathway enrichments emerge as promising biomarkers and therapeutic targets. Our findings advance the molecular understanding of this aggressive equine cancer, laying a foundation for precision diagnostic and therapeutic strategies.

## Author Contributions

**Paitoon Srimontri:** conceptualisation; investigation; methodology; visualisation; editing; project administration. **Nawarus Prapaiwan:** conceptualisation; investigation; editing; project administration. **Thanapon Chotikaprakal:** conceptualisation; investigation; validation; methodology; visualisation. writing – original draft; visualisation; writing – review and editing. **Rangsima Sujittosakul**, **Nichapat Iamkaewprasert**, **Jiraschaya Piputwat**, **Puetta Isama‐al**, **Thanutchanok Munkongdee** and **Petchpailin Yanyongsirikarn:** investigation; methodology. **Narumon Phaonakrop:** investigation; methodology. **Sittiruk Roytrakul** and **Amornthep Kingkaw:** conceptualisation; investigation; writing – original draft; validation; methodology; visualisation; writing – review and editing; project administration; data curation; supervision; formal analysis; resources. **Wanwipa Vongsangnak:** conceptualisation; investigation; writing – original draft; validation; methodology; visualisation; writing – review and editing; project administration; data curation; supervision; formal analysis; resources. **Parichart Tesena:** conceptualisation; investigation; writing – original draft; validation; methodology; visualisation; writing – review and editing; project administration; data curation; supervision; formal analysis; resources; funding acquisition.

## Funding

This work was supported by the Mahidol University's Strategic Research Fund: 2023 (MU‐SRF‐RS‐14A/66) and the Faculty of Veterinary Science, Mahidol University.

## Disclosure

No cell lines were used in this study.

## Ethics Statement

This study was conducted in accordance with ethical guidelines and approved by the Institutional Animal Care and Use Committee (IACUC) of the Faculty of Veterinary Science, Mahidol University, Thailand (approval number: MUVS‐2024‐02‐09). Informed consent was obtained from all horse owners prior to participation. No cell lines were used in this study, which relied on the direct analysis of equine tissue biopsy samples; therefore, cell line validation was not applicable.

## Conflicts of Interest

The authors declare no conflicts of interest.

## Supporting information


**File S1:** Signalments, clinical classification and EMN category of grey horses.
**File S2:** List of unique proteins from differentially expressed proteins analysis under Wald test in DESeq2 between normal vs. early stage(N vs. E), normal vs. severe (N vs. S) and early vs. severe (E vs. S) EMN stages of tissue biopsy sample.

## Data Availability

The raw MS/MS spectra data are available in ProteomeXchange: JPST003894 (https://repository.jpostdb.org/preview/1084520173685e53ef3d765, Accession key 4115) and PXD065534. The datasets used and/or analysed during the current study are available from the corresponding author on reasonable request.
